# The Impact of Rootstock on “Big Top” Nectarine Postharvest Concerning Chilling Injury, Biochemical and Molecular Parameters

**DOI:** 10.3390/plants13050677

**Published:** 2024-02-28

**Authors:** Aimar Navarro, Rosa Giménez, Jesús Val, María Ángeles Moreno

**Affiliations:** 1 Department of Pomology, Estación Experimental de Aula Dei—Consejo Superior de Investigaciones Científicas (EEAD-CSIC), P.O. Box 13034, 50080 Zaragoza, Spain; aimarnavarro@eead.csic.es (A.N.); rosagsoro@eead.csic.es (R.G.); 2 Department of Genetics and Plant Production, Estación Experimental de Aula Dei—Consejo Superior de Investigaciones Científicas (EEAD-CSIC), P.O. Box 13034, 50080 Zaragoza, Spain; 3 Department of Plant Biology, Estación Experimental de Aula Dei—Consejo Superior de Investigaciones Científicas (EEAD-CSIC), P.O. Box 13034, 50080 Zaragoza, Spain; jesus.val@csic.es

**Keywords:** antioxidants, bleeding, browning, cold storage, enzymatic activity, fruit quality, gene expression

## Abstract

Peaches and nectarines have a short shelf life even when harvested at appropriate physiological maturity. Market life is increased by storage at low temperatures. However, chilling injury symptoms can appear, causing physiological disorders and limiting shipping potential. The rootstock effect on the post-harvest quality has hardly been explored. Thus, the principal aim of this work was to study the influence of seven different *Prunus* rootstocks on the “Big Top” nectarine cv, considering harvest and post-harvest quality parameters and their correlation with chilling injury disorders. Basic fruit quality traits, individual sugars and organic acids analyzed by HPLC and other biochemical compounds such as relative antioxidant capacity, total phenolics content, flavonoids, anthocyanins, vitamin C and related enzyme activities (PAL, POD, PPO) were considered. In addition, correlations with possible candidate genes for chilling injury (CI) tolerance were searched by qPCR. Although a low susceptibility to CI symptoms has been found in “Big Top”, rootstocks “PADAC 9902-01”, “PADAC 99-05” and “ReplantPAC” exhibited lower CI symptoms. A statistically significant influence of the evaluated rootstocks was found concerning the parameters of this study. Phenols and anthocyanins seem to be important parameters to be considered in the prevention of chilling injury disorders. Moreover, *PAL1*, *PPO4*, *PG2* and *LDOX* genes relative expressions were positively associated with chilling injury susceptibility. This study opens new perspectives for understanding peach fruit adaptation and response to cold storage temperatures during the post-harvest period.

## 1. Introduction

Peaches (*Prunus persica*) and nectarines (*P. persica* var. nectarine) are closely related and belong to the *Rosaceae* family. Currently, peach and nectarine world production stands at 25 million tons. Spain is the second largest producer in the world, with a production of 1.2 million tons in 2021 [1], and the first exporter. New sweet and full-colored nectarines have increased their importance in Spain during the last two decades, such as “Big Top”. It is a clingstone nectarine cultivar from the USA with a peculiar characteristic. Although it is considered a slow-melting type, it resembles, at harvest, a stony hard fruit, with high firmness, but then it slowly melts at full ripening [2].

Peaches and nectarines are climacteric fruits and therefore, if they have been collected in the suitable state of maturity, they continue ripening once harvested from the tree. However, their rapid ripening results in a short market life. Refrigeration is the most common method employed to increase commercial prospects. However, low temperatures can negatively affect fruit quality and produce physiological alterations well known as chilling injury (CI) symptoms, limiting their preservation [3]. Thus, comprehending and preventing CI mechanisms are of a great economic and scientific interest.

The main chilling injury symptoms are browning, mealiness, bleeding and leatheriness. Browning symptoms have been associated with changes in the membrane’s permeability, putting into contact phenolic compounds and oxidative enzymes such as polyphenol oxidase (PPO) and peroxidase (POD) [4]. Mealiness is produced by the separation of parenchymatic cells due to an alteration in cell wall water retention capacity. It has been attributed to an increase in pectin methyl esterase (PME) activity and an inhibition of endo-Polygalacturonase (PG) activity, causing poor texture and high levels of insoluble pectins [4]. Phenylalanine Ammonia Lyase (PAL) is an important enzyme involved in the phenylpropanoid pathway and related with the phenolic compounds synthesis [5]. PAL may also be implicated in CI disorders since total phenolic content has been previously associated with browning potential [4]. Bleeding is the reddening of the flesh, produced by anthocyanin accumulation mainly due to cyanidin 3-glucoside. Unlike other symptoms, red pigment accumulation does not affect fruit taste [6]. Leatheriness is characterized by a tough and hard texture similar to leather. Chilling injury symptoms mainly depend on post-harvest temperature and storage period. The expression of these disorders develops faster and more strongly when fruits are stored at 5 °C than at 0 °C [3].

There have been significant efforts toward the identification of genes underlying CI symptoms. Pavez et al. [7] showed an increase in components of stress after cold storage, such as gene encoding superoxide dismutase (SOD). Cao et al. [8] showed that peaches that suffered from mealiness up-regulated *PME* and down-regulated *PG*. Genero et al. [9] showed that *Expansin* (*EXP*) expression increased with ripening and was correlated with mealiness. An anthocyanins biosynthesis pathway gene, *Leucoanthocyanidin Dioxygenase* (*PpLDOX*), was co-located with the major QTL for browning on linkage group 5 [10].

Rootstock affects scion fruit composition [11], and therefore we hypothesize that it affects the fruit preservation potential. Rootstocks offer a wide range of possibilities for peach production because different species and hybrids from the *Prunus* genus can be used. New rootstocks inducing less tree vigor, better fruit quality and higher yield efficiency are currently under development or available on the market [12,13].

Although several studies have been performed to improve our knowledge about the biochemical and molecular basis of CI, most of them were carried out with different cultivars grafted on the same rootstock. Consequently, this study aims to assess the effect of new and some of the most relevant *Prunus* rootstocks on chilling injury symptoms and post-harvest fruit quality. Previous reports have indicated a significant rootstock influence on “Big Top” at harvest, particularly regarding agronomical performance and biochemical compounds, such as sugars and antioxidants in fruits [11,12,14,15]. Antioxidants improve the health-promoting potential of fruits, counteract oxidative stress, suppress ROS formation and inhibit the oxidation of other molecules [16]. However, although rootstock effect on fruit quality has been studied at harvest, rootstock effect on post-harvest quality in fruits has hardly been explored [17,18]. To the best of our knowledge, the present assay is the first study reporting on peaches’ post-harvest rootstock effect in CI disorders related to biochemical and molecular parameters evaluated during two consecutive years.

To fulfill the main objective, soluble solids content (SSC), titratable acidity (TA), flesh firmness (FF), sugars and organic acids profile, relative antioxidant capacity (RAC), total phenolics content (TPC), total flavonoids content (TFC), anthocyanin content (AC), enzymatic activities of the phenylalanine ammonia lyase (PAL), polyphenol oxidase (PPO) and Peroxidase (POD) were evaluated both at harvest and post-harvest conditions. In addition, differential expressions of candidate genes related to CI disorders were also analyzed. We aim to determine which biochemical parameters are influenced by both the rootstock and the cold storage treatment plus two days of shelf life, to establish the most important parameters for the selection of rootstocks including a better post-harvest quality and lower CI symptoms.

## 2. Results and Discussion

### 2.1. Chilling Injury Symptoms

**Figure 1:**
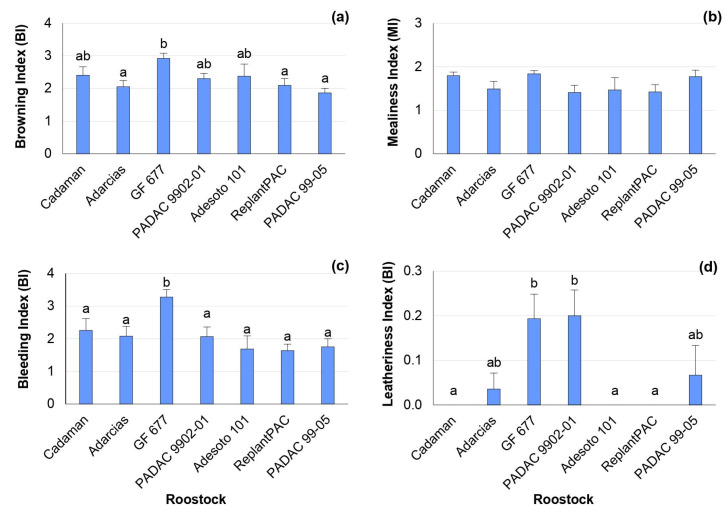
Rootstock influence on chilling injury symptoms of “Big Top” fruits. Means showed the average for 2020 and 2021 seasons after 28 days of cold storage at 5 °C plus 2 days of shelf life (28 + 2 d). Bars represent the standard errors of the means. Means with different letters are significantly different (*p* ˂ 0.05) according to the Duncan’s test. (**a**) Browning. (**b**) Mealiness. (**c**) Bleeding. (**d**) Leatheriness.

The “Big Top” nectarine did not suffer severe CI symptoms after 28 days of cold storage at 5 °C plus 2 days of shelf life (28 + 2 d) (Figure 1), compared to most studied cultivars from a large collection of peaches [19]. In fact, it has been found that nectarines have a better resilience to storage conditions than peaches and specially the “Big Top” nectarine due to its specific properties [2,20]. Nevertheless, significant differences were found among rootstocks for browning, bleeding and leatheriness symptoms, with mean percentage differences among rootstocks of 21.6, 17.2 and 30.6%, respectively. The rootstock that induced the highest browning index was the peach–almond hybrid “GF 677”, although it did not differ significantly from “Cadaman”, “Adesoto 101” and “PADAC 9902-01” (Figure 1a). The highest bleeding index was found for “GF 677” (Figure 1c). Additionally, the highest leatheriness index was found for the peach–almond hybrids “GF 677” and “PADAC 9902-01” (Figure 1d), although they did not differ from “Adarcias” and “PADAC 99-05”. Hence, “ReplantPAC” was the rootstock that induced, in general, lower CI symptoms, having a plum–almond genetic background.

The chilling injury symptoms were significantly correlated between them. Thus, browning was positively correlated with mealiness (r = 0.573 *) and bleeding (r = 0.872 **). Mealiness was also positively correlated with bleeding (r = 0.635 *). Other studies have previously reported significant correlations between mealiness and browning [6], as well as between mealiness and bleeding [21].

### 2.2. Basic Fruit Quality

Basic fruit quality presented significant differences between harvest and post-harvest conditions (Table 1 and Table S1). Given the experimental design employed in this study, it is important to note that the comparisons made between the harvest and 28 + 2 d time points do not make it possible to ascertain whether observed changes are attributable to the chilling treatment or to subsequent shelf-life conditions. The flesh firmness of the fruits sharply decreased during post-harvest, as previously reported [17]. After 28 + 2 d, firmness was negatively correlated with the mealiness symptoms (r= −0.638 *) as this disorder is characterized by a woolly texture [3]. At harvest, SSC was the only parameter correlated with yield (r = −0.350 *). SSC slightly increased during the treatment. In contrast, TA, in general, significantly decreased according to Navarro et al. [19]. Lower values were also observed for TA and firmness after 21 days of cold storage plus 3 days of shelf life in the “Maciel” peach cultivar grafted on different rootstocks [18].

### 2.3. Sugars and Organic Acids Profiles

**Figure 2:**
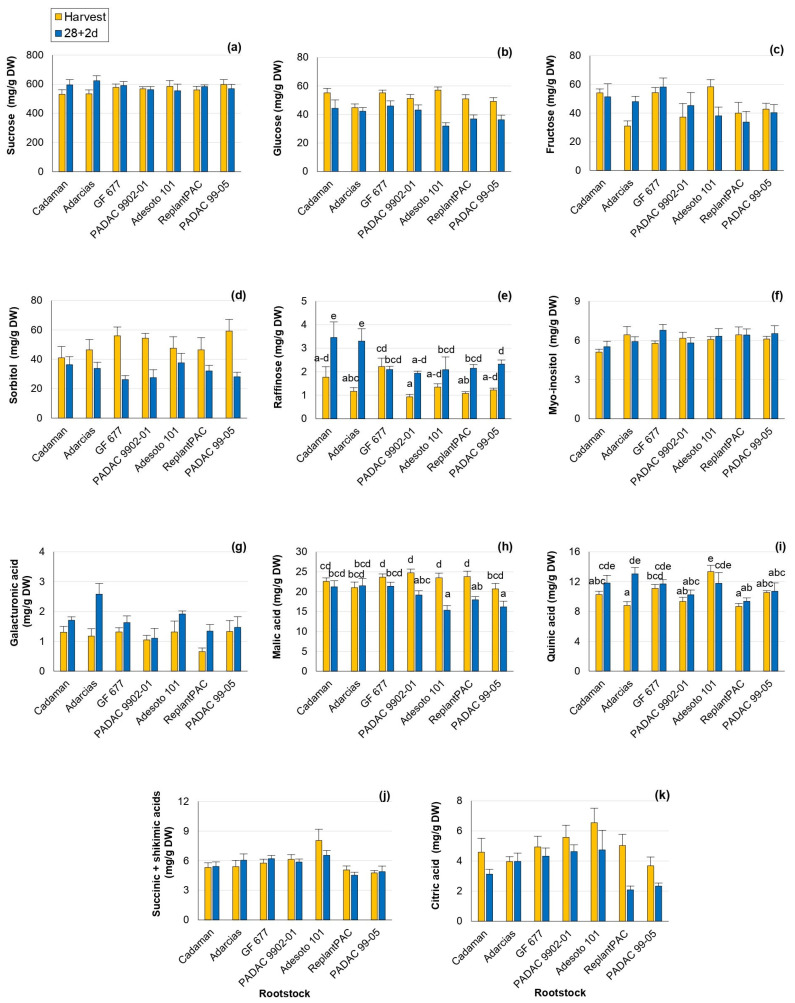
Rootstock influence on sugars and organic acids of “Big Top” fruits. (**a**) Sucrose (mg/g DW). (**b**) Glucose (mg/g DW). (**c**) Fructose (mg/g DW). (**d**) Sorbitol (mg/g DW). (**e**) Raffinose (mg/g DW). (**f**) Myo-Inositol (mg/g DW). (**g**) Galacturonic acid (mg/g DW). (**h**) Malic acid (mg/g DW). (**i**) Quinic acid (mg/g DW). (**j**) Succinic + Shikimic acids (mg/g DW). (**k**) Citric acid (mg/g DW). Means showed the average for 2020 and 2021 seasons at harvest and after 28 days of cold storage at 5 °C plus 2 days of shelf life (28 + 2 d). Bars represent the standard errors of the means. Means with different letters are significantly different (*p* ˂ 0.05) according to Duncan’s test.

The sugars and organic acids profiles play an important role in peach taste perception. Changes in the individual sugars and organic acids have been reported with cold exposure in comparison with harvest values [22,23], showing roles as osmoregulators, cryoprotectants and signaling molecules. As in the mentioned studies, changes in individual sugars and organic acids were found in this work (Tables S2 and S3). Sucrose, glucose, fructose and sorbitol were the main individual sugars found, as previously reported [11]. Significant differences among rootstocks were found for fructose, raffinose and galacturonic acid. However, this effect was not consistent between years for fructose and raffinose (Table 1). **Sucrose** was the most abundant individual sugar and it has been repeatedly associated with chilling injury tolerance [23] and processes of stabilization of membranes. Thus, rootstocks that induce higher sucrose content should improve fruit conservation. **Glucose** content significantly decreased after 28 + 2 d (Figure 2b). Reduced levels of glucose have been associated with lower CI symptoms caused by sucrose accumulation with a protectant role [24]. In accordance, after 28 + 2 d, glucose was positively correlated with bleeding (r = 0.534 *). **Fructose** levels varied significantly among rootstocks, with a percentage difference of 12.5% (Figure 2c). “Adesoto 101” induced the highest values of fructose at harvest conditions and “GF 677” at post-harvest. Moreover, fructose levels after 28 + 2 d were positively correlated with browning (r = 0.557 *), mealiness (r = 0.665 **) and bleeding (r = 0.592 *) symptoms, in agreement with Wang et al. [24]. **Sorbitol** has been proposed to be implicated in the response to chilling stress as an osmotic protectant [22]. In the present work, sorbitol significantly decreased after 28 + 2 d, especially in “GF 677”, “PADAC 9902-01” and “PADAC 99-05” (Figure 2d). In contrast, **raffinose** values increased in comparison with harvest for all rootstocks, with the exception of “GF 677” (Figure 2e), one of the rootstocks inducing higher browning, bleeding and leatheriness symptoms. In fact, raffinose-increasing levels have been previously reported to be induced by cold storage with an important role in CI tolerance, acting as antioxidant and stabilizing the membranes system [22]. “ReplantPAC” and “Cadaman” seemed to induce the highest **myo-inositol** levels (Figure 2f) and they were among the rootstocks with lower CI symptoms. Myo-inositol levels have been negatively associated with susceptibility to CI [22]. **Galacturonic acid** values significantly differed among rootstocks, with a percentage difference of 17.5%, and they increased after 28 + 2 d (Figure 2g), with the highest increase observed in “Adarcias”. This was probably produced during shelf life as a consequence of maturation, when a dramatic increase in polygalacturonase and galacturonic acid was observed in “Big Top” [2]. Galacturonic acid increase has been associated with lower susceptibility to mealiness symptoms in other studies [22].

In the case of organic acids, **malic acid** was the most abundant organic acid and it has been suggested as the greater contributor to acidity in peaches along with citrate [25]. Hence, malic (Figure 2h) and citric (Figure 2k) acids were significantly affected by rootstock, with a difference among them of 5.8 and 15.1%, respectively, and they significantly decreased after 28 + 2 d (Table 1). After 28 + 2 d, “Adarcias” induced the highest level of malic acid, although it did not differ from “GF 677” and “Cadaman”. The malic acid was positively correlated with bleeding after 28 + 2 d (r = 0.630 *). Bleeding, as a consequence of tissue senescence, has been previously associated with organic acids [3]. The **citric acid** significantly decreased, in general, with storage (Figure 2k) in accordance with Brizzolara et al. [22] who reported that low-acid cultivars, such as “Big Top”, showed citrate degradation during cold storage. In contrast to malic and citric acids, the **quinic acid** levels increased, in general, after 28 + 2 d (Figure 2i). Quinic acid is a precursor of chlorogenic acids. A low accumulation of chlorogenic acids has been associated with the repression of reddening and browning in the Japanese “Okayama” peach cv. [26]. A difference of 8.1% was observed among rootstocks, and “Adesoto 101” induced the highest level at harvest and “Adarcias” after 28 + 2 d. The sum of **succinic + shikimic acids** presented significant differences of 10.6% among rootstocks, and “Adesoto 101” induced the highest levels at harvest and after 28 + 2 d (Table 1 and Figure 2c).

All the studied acids significantly differed among rootstocks. Moreover, this effect was consistent between years (Table 1 and Table S3). “Adesoto 101” was the rootstock that induced higher TA, quinic, succinic + shikimic and citric acids. Hence, it can increase the sweetness perception of “Big Top”, inducing higher contents of organic acids in good agreement with Font i Forcada et al. [13] and Baccichet et al. [25]. Surprisingly, it was also one of the rootstocks that induced lower malic acid levels after 28 + 2 d.

### 2.4. Antioxidants and Related Enzymatic Activities (PAL, POD, PPO)

“Big Top” nectarine cv. has been characterized as a low antioxidant capacity cultivar [2]. Consequently, it could be desirable to increase antioxidant levels in “Big Top” fruits to induce a better fruit quality in terms of nutrition. It is well known that rootstock can affect antioxidant capacity [11,12,15] and enzymes related with antioxidant compounds synthesis [5] and oxidation that, in turn, are involved in chilling injury.

In the present study, all the antioxidant compounds exhibited notable sensitivity to the rootstock effect (Table 1), with mean percentage differences among rootstocks: relative antioxidant capacity (RAC) by 14.1%, total phenolics content (TPC) by 14.8%, total flavonoids content (TFC) by 28.5%, anthocyanins content (AC) by 33.2% and ascorbic acid (AsA) by 10.5%. In addition, this effect was consistent between years, except for RAC and TFC. Significant differences between harvest and 28 + 2 d were only observed for anthocyanin and vitamin C contents (Table S4).

**Figure 3:**
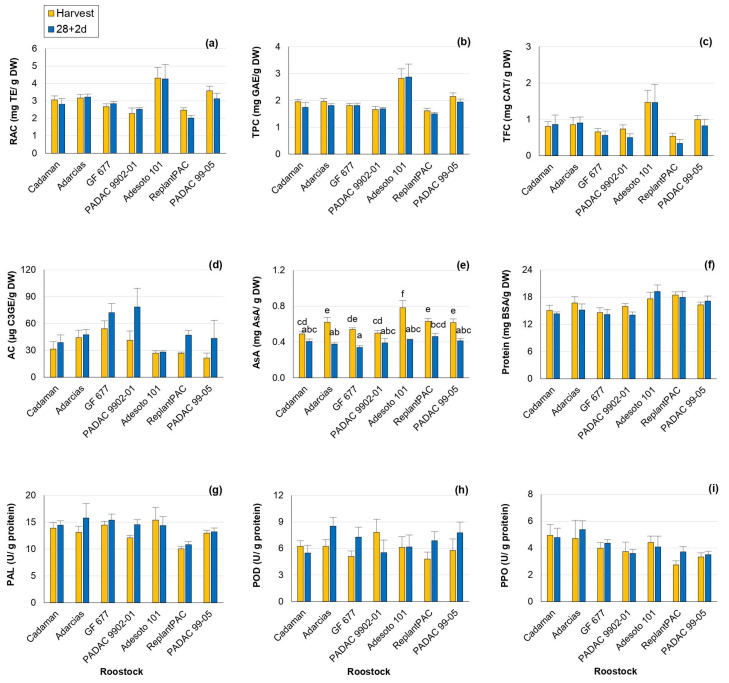
Rootstock influence on antioxidants and enzyme activity of “Big Top” fruits. (**a**) RAC (mg TE/g DW). (**b**) TPC (mg GAE/g DW). (**c**) TFC (mg CAT/g DW). (**d**) AC (µg C3GE/g DW). (**e**) Vitamin C (mg AsA/g DW). (**f**) Protein content (mg BSA/g DW). (**g**) PAL activity (U/g protein). (**h**) POD activity (U/g protein). (**i**) PPO activity (U/g protein). Means showed the average for 2020 and 2021 seasons at harvest and after 28 days of cold storage at 5 °C plus 2 days of shelf life (28 + 2 d). Bars represent the standard errors of the means. Means with different letters are significantly different (*p* ˂ 0.05) according to Duncan’s test.

At harvest and post-harvest, the plum “Adesoto 101” rootstock induced the highest RAC (Figure 3a), TPC (Figure 3b) and TFC (Figure 3c), the main antioxidants in peach, and this performance was consistent between years (Table S4). In contrast, the lowest values were induced by “ReplantPAC”, “PADAC 9902-01” and “GF 677”. With the exception of “ReplantPAC”, plum-based rootstocks showed, in general, higher RAC and TPC, as reported by Font i Forcada et al. [11]. There was a tendency for antioxidant capacity and phenols to decrease after 28 + 2 d. TPC decrease may be due to phenolic compounds oxidation associated with browning [16].

Flavonoids are the most common phenolic compounds. The rootstock that induced the highest TFC, both at harvest and post-harvest, was also “Adesoto 101”, followed by “PADAC 99-05”, although the latter did not differ from the rest of rootstocks (Figure 3c). RAC was highly and directly correlated with TPC both at harvest (r = 0.959 **) and at 28 + 2 d (r = 0.924**). TPC was also positively correlated with flavonoids both at harvest (r = 0.920 **) and at 28 + 2 d (r = 0.817 **). Moreover, it is interesting to note that “Adesoto 101” was the rootstock exhibiting, in general, higher RAC, TPC and TFC, as well as quinic and succinic + shikimic acids.

Anthocyanins are flavonoids end-products with roles in plant resistance against biotic and abiotic stresses [27]. In addition, anthocyanin accumulation has been associated with bleeding [28]. In this work, anthocyanin content (AC) was statistically different among rootstocks and treatments. AC increased after 28 + 2 d (Figure 3d), probably as a bleeding consequence. “GF 677” and “PADAC 9902-01” were the rootstocks that increased AC the most in relation to harvest.

Significant differences among rootstocks were also reported for vitamin C (AsA) levels in “Big Top”, in agreement with Font i Forcada et al. [11] and Reig et al. [15]. In general, plum-based rootstocks induced higher AsA values compared with peach and almond-based rootstocks. “Adesoto 101” and “ReplantPAC” induced the highest values both at harvest and post-harvest, although they did not differ from “PADAC 99-05”, “GF 677” and “Adarcias”, at harvest, and from “PADAC 99-05”, “PADAC 9902-01” and “Cadaman”, at post-harvest (Figure 3e). Vitamin C decreased significantly for most rootstocks after 28 + 2 d. In a recent study [29], the exogenous application of AsA significantly alleviated CI symptoms in “Florida Prince” peaches. In accordance with this, ascorbic acid was negatively correlated with bleeding (r = −0.568 *) and leathering (r = −0.794 *) symptoms after 28 + 2 d in the present work.

Enzyme activities (PAL, POD and PPO) and protein content were affected by year and rootstock (Table 1 and Table S5; Figure 3f–i). Significant differences among rootstocks of 8.7, 8.1 and 13.8% were observed for protein content, PAL and PPO activities, respectively.

In the case of PAL average values, the rootstock “ReplantPAC” induced the lowest content (Figure 3g, Table S5). PAL is an important enzyme involved in the phenylpropanoid pathway and therefore, it is related to the synthesis of phenols as anthocyanins, flavonols and other secondary metabolites [30]. PAL activity could be increased with stress (as cold storage), also causing an increase in phenolic content. Thus, PAL activity was positively correlated with RAC both at harvest (r = 0.740 **) and at 28 + 2 d (r = 0.557 *); as well as with TPC at harvest (r = 0.756 **) and with TFC both at harvest (r = 0.802 **) and at 28 + 2 d (r = 0.668 **).

PPO is the main enzyme responsible for browning, although POD seems to be also correlated with browning symptoms. They produce the oxidation of phenols leading to the formation of brown polymers [4]. For both years, “Adarcias” and “ReplantPAC” were the rootstocks exhibiting the higher and lower values, respectively (Table S5). However, “Adarcias” did not differ significantly from “Cadaman”, “GF 677”, “PADAC 9902-01” and “Adesoto 101”. “ReplantPAC” only differed significantly from “Adarcias” and “Cadaman” (Figure 3i). In general, POD and PPO showed a tendency to increase their activities after 28 + 2 d (Table S5).

POD and PPO enzymatic activities were positively correlated between them both at harvest (r = 0.596 *) and after 28 + 2 d (r = 0.597 *). The TFC was negatively correlated with the POD enzymatic activity after 28 + 2 d (r = −0.551*). Moreover, POD activity at harvest was negatively correlated with mealiness symptoms at 28 + 2 d (r = −0.723 **).

### 2.5. Gene Expression Analysis

*EST* (No. DY652828) was the most stable reference gene with M = 0.143, followed by *RP II* (M = 0.282), *eIF* (M = 0.325), *ACT1* (M = 0.362) and *TEF2* (M = 0.400). Consequently, *EST* was used as the reference gene. The “GF 677” expression value at harvest was used as control because it is the most widely used peach–almond hybrid rootstock in the Mediterranean region [13,14].

**Figure 4:**
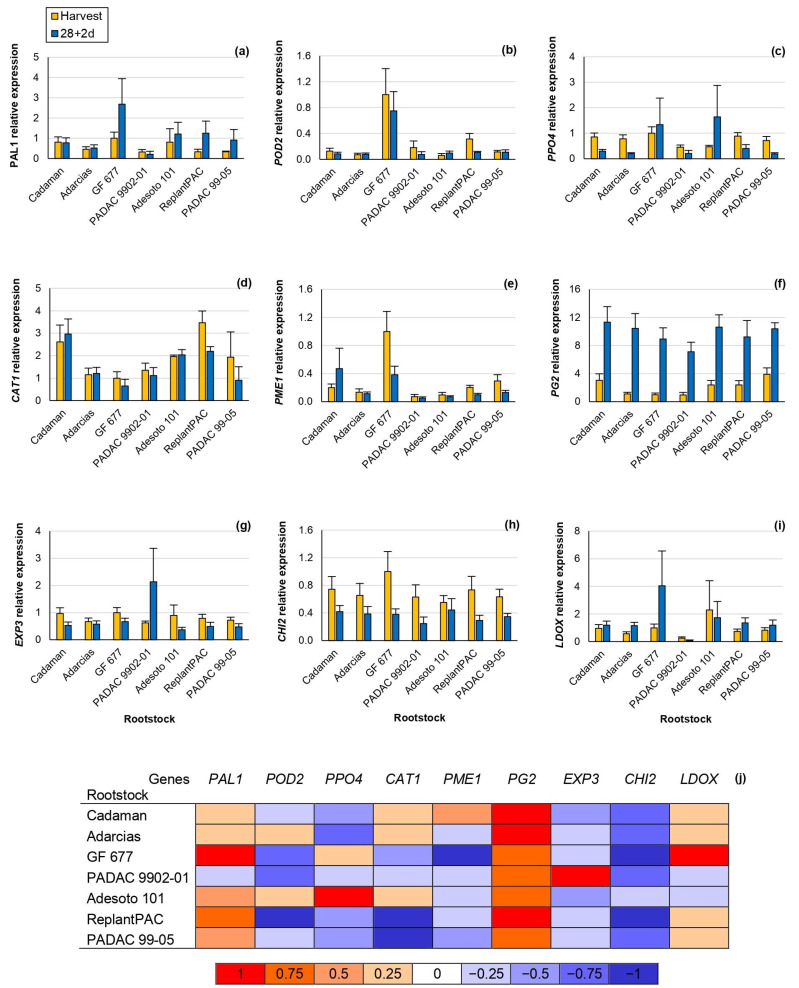
RT-PCR analysis of key genes involved in chilling injury in the “Big Top” cv. budded on different *Prunus* rootstocks. Data were normalized using “GF 677” expression at harvest as control. Means showed the average for 2020 and 2021 seasons at harvest and after 28 days of cold storage at 5 °C plus 2 days of shelf life (28 + 2 d). Bars represent the standard errors of the means. Means with different letters are significantly different (*p* ˂ 0.05) according to Duncan’s test. (**a**) *Phenylalanine Ammonia Lyase 1* (*PAL1*). (**b**) *Peroxidase 2* (*POD2*). (**c**) *Polyphenol Oxidase 4* (*PPO4*). (**d**) *Catalase 1* (*CAT1*). (**e**) *Pectin Methylesterase 1* (*PME1*). (**f**) *Polygalacturonase 2* (*PG2*). (**g**) *Expasin 3* (*EXP3*). (**h**) *Chalcone Synthase 2* (*CHI2*). (**i**) *Leucoanthocyanidin Dioxygenase* (*LDOX*). (**j**) Heat map. The different colors represent the increase or decline in the expression at 28 + 2 d in comparison with the harvest.

In this study, significant differences were observed concerning the relative gene expression among rootstocks, for the genes *POD2*, *CAT1*, *PME1 and PG2* (Table 2). Moreover, this effect was consistent between years, except for *CAT1*. Significant differences were observed between harvest and 28 + 2 d, for *PPO4*, *CAT1, PG2*, *EXP3* and *CHI2* (Figure 4).

*Phenylalanine Ammonia Lyase 1* (*PAL1*) was positively correlated at harvest with browning (r = 0.672 **), mealiness (r = 0.731 **) and bleeding (r = 0.609 **). Hence, it seems that fruits suffering some kind of stress at harvest may also be suffering from higher CI symptoms at post-harvest.

*Peroxidase 2* (*POD2*) codifies a peroxidase that seems to be also correlated with the browning symptoms [4]. In accordance, the *POD2* relative expression of “GF 677”, one of the rootstocks inducing higher chilling injury symptoms, was at least three-fold higher than the rest of the rootstocks, both at harvest and after 28 + 2 d (Figure 4b).

*Polyphenol Oxidase 4* (*PPO4*) relative expression, in general, significantly decreased after 28 + 2 d, although “GF 677” and “Adesoto 101” increased it (Figure 4c). *PPO4* relative expression and browning symptoms were slightly but significantly correlated at 28 + 2 d (r = 0.472 *). Similarly, Wang et al. [27] reported that the *PPO* relative expression and browning symptoms were correlated in the “Yuhua” peach cv. after different periods of cold storage.

*Catalase 1* (*CAT1*) relative expression highly differentiates among rootstocks, both at harvest and 28 + 2 d, showing the highest values for “ReplantPAC”, although it did not significantly differ from “Cadaman” and “Adesoto 101” (Figure 4d). They induced, in general, a two-fold higher expression than the rest of the rootstocks. Toivonen and Brummell [31] reported that catalase reduced oxidative damage delaying the occurrence of CI by increasing its expression. In good agreement, “ReplantPAC” showed the highest expression at harvest and was among the rootstocks exhibiting lower CI symptoms. Furthermore, the down-regulation of *CAT* has been associated with higher cold stress sensibility [20].

Pectin methyl esterase (PME) and polygalacturonase (PG) enzymes cause firmness reduction by pectin degradation. PME acts during all peach fruit life while PG is considered as the enzyme with the greatest contribution to softening [8]. At harvest, the *PME1* relative expression of “GF 677” was approximately three-fold higher than “PADAC 99-05”; five-fold higher than “Cadaman”, “Adarcias” and “ReplantPAC”; and ten-fold higher than “PADAC 9902-01” and “Adesoto 101” relative expressions. *PG2* significantly increased after cold treatment (Figure 4j). Mealiness has been attributed to the increase in PME activity and the inhibition of PG activity causing insoluble pectins [8]. These changes may also be attributed to maturation during shelf life, as “Big Top” hardly suffers slight mealiness symptoms. “Big Top” melts at a slow speed, with low PG transcription levels at harvest and during ripening, while a dramatic increase is produced, leading to fruit melting [2]. Thus, *PME1* and *PG2* were significantly and negatively correlated with firmness (r = −0.479 * and r = −0.692 **, respectively) after 28 + 2 d. Moreover, the increase in *PG2* after 28 + 2 d was positively correlated with mealiness (r = 0.698 **).

Expansin is a cell-wall-modifying protein that has been related with the mealy texture [9]. In this study, *EXP3* relative expression significantly decreased after 28 + 2 d (Figure 4g,j), with the exception of “PADAC 9902-01” which induced a three-fold higher expression after 28 + 2 d than at harvest.

*Chalcone synthase 2* (*CHI2*) highly decreased after 28 + 2 d (Figure 4h,j). *CHI2* codifies for a chalcone synthase, involved in flavonoids synthesis. Accordingly, the same tendency was shown for TFC, decreasing after 28 + 2 d (Figure 3c). Thus, *CHI2* relative expression was significantly correlated with TFC (r = 0.549 *). In general, a two-fold lower expression was observed after 28 + 2 d than at harvest for all rootstocks with the exception of “PADAC 04-03” decreasing more than three-fold.

Finally, leucoanthocyanidin dioxygenase (LDOX) is one of the multi-enzyme complexes implicated in anthocyanins biosynthesis. It was correlated with PAL enzymatic activity (r = 0.546 *) as both enzymes are implicated in the same pathway. Moreover, after 28 + 2 d, it was positively correlated with raffinose values (r = 0.563 *), according to previous studies that correlated several soluble sugars increasing (glucose, sucrose, sorbitol and fructose) with *LDOX* expression [27]. The *LDOX* relative expression, both at harvest and at 28 + 2 d, was significantly correlated with browning (r = 0.544 * and r = 0.559 *, respectively), as previously reported [10].

Although previous studies correlated all these genes with chilling injury symptoms [20,31,32], they studied cultivars more susceptible to CI than “Big Top” and it could help to explain why those correlations were not confirmed in some cases. Furthermore, the inclusion of two days of shelf life may have contributed to the differences observed. The differences also suggest that different mechanisms act when comparing susceptible and non-susceptible cultivars to cold storage alterations.

We must highlight that *PAL1* positively correlated at harvest with browning, mealiness and bleeding; *PPO4* positively correlated at 28 + 2 d with browning; *PG2* positively correlated at 28 + 2 d with mealiness; and *LDOX* positively correlated at 28 + 2 d with browning.

### 2.6. Principal Component Analysis (PCA)

**Figure 5:**
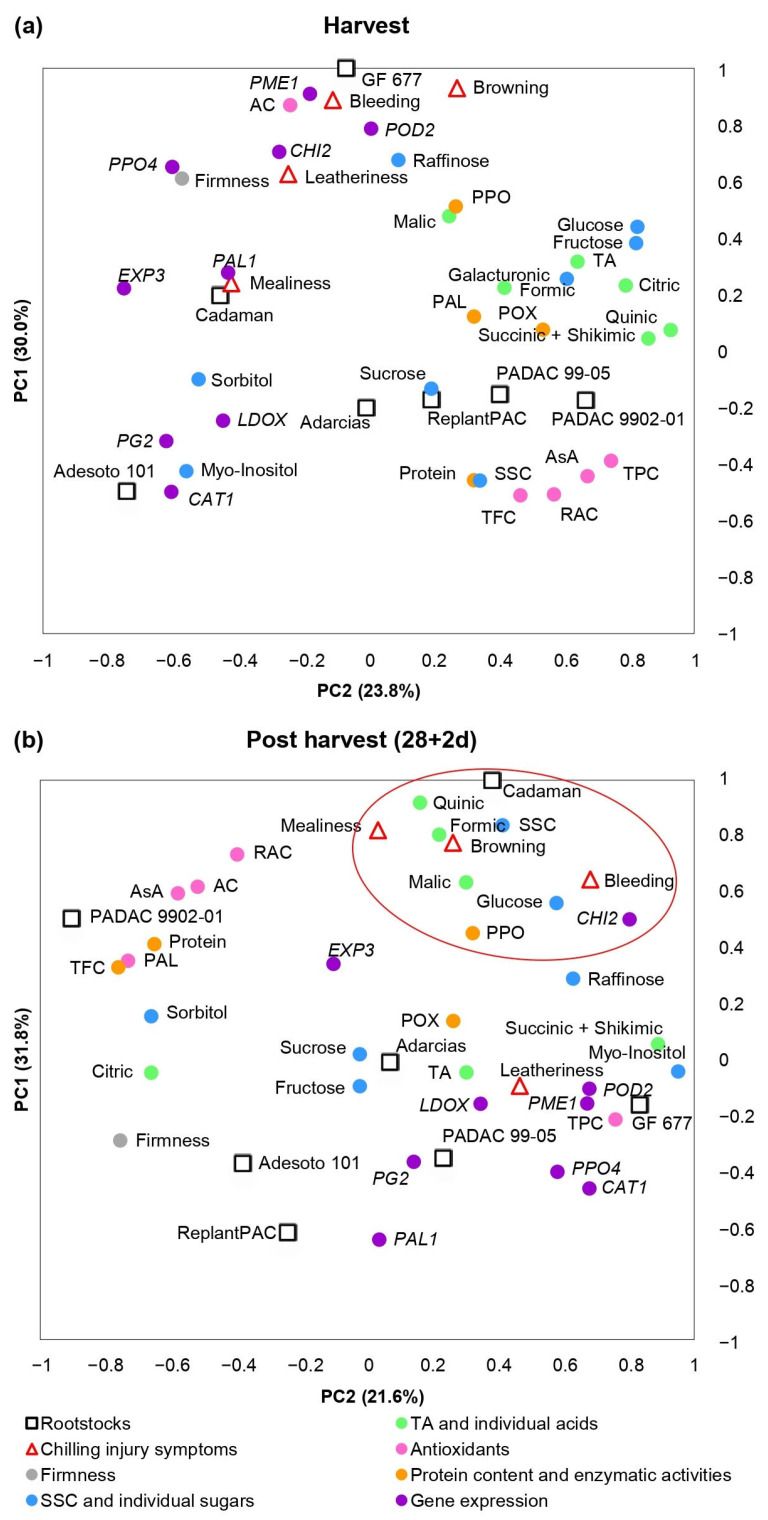
Principal component analysis (PCA) of chilling injury symptoms, basic quality parameters, biochemical traits and gene expressions at harvest (**a**) and after 28 days at 5 °C plus 2 days of shelf life (**b**) in “Big Top” cv. budded on different *Prunus* rootstocks. Red circle highlights the parameters close to chilling injury symptoms. Average data of 2020 and 2021 years were used.

As it has been previously explained, significant differences among rootstocks and treatments were found for the biochemical parameters and genes considered in this study. Moreover, significant correlations were found between them and chilling injury symptoms. A principal component analysis (PCA) was performed in order to clarify the contribution of harvest (Figure 5a) and post-harvest (Figure 5b) values and chilling injury symptoms to the variability among the different rootstocks.

Figure 5a showed parameters evaluated at harvest and CI symptoms observed at 28 + 2 d. More than 53% of the observed variance could be explained by the first two components. PC1 differentiated “GF 677” and “Cadaman” (peach–almond and *P. persica* × *P. davidiana*-based rootstocks, respectively) from “Adarcias”, “PADAC 9902-01” (both peach–almond rootstocks) and plum-based rootstocks “PADAC 99-05” (plum x peach–almond hybrid), “Adesoto 101” (hexaploid plum) and ReplantPAC (plum–almond hybrid). PC2 differentiated “GF 677”, “Cadaman”, “Adarcias” and “Adesoto 101” from “PADAC 9902-01”, “PADAC 99-05” and “ReplantPAC”. The rootstocks on the positive side of the PC1 induced, in general, higher CI symptoms as well as *PME1*, *CHI2*, *PPO4*, *EXP3* and *PAL1* relative expressions, higher PAL, POD and PPO enzymatic activities, higher acids and higher glucose, fructose, raffinose and galacturonate concentrations. On the other hand, in the negative side of the PC1, the rootstocks induced higher levels of antioxidants (TPC, TFC, RAC), sucrose, sorbitol, myo-inositol, SSC and *PG2*, *LDOX* and *CAT1*-relative expressions.

Figure 5b shows the parameters after 28 days of cold storage plus 2 of shelf life (28 + 2 d). More than 53% of the observed variance could be explained by the first two components. The rootstocks on the negative side of the PC2 induced, in general, higher CI symptoms and, as seen in Figure 5a, higher *PME1*, *CHI2*, *PPO4* and *PAL1*-relative expressions, POD and PPO enzymatic activities and, in general, higher organic acids. Differentially, after 28 + 2 d, the rootstocks on the negative side of the PC2 induced higher *CAT1*, *PG2*, *LDOX* and *POD2*-relative expression and, in general, higher sugars. On the other hand, in the positive size of the PC2, the rootstocks induced higher firmness, higher levels of antioxidants (TPC, TFC, RAC), sucrose, sorbitol, fructose and *EXP3*-relative expression.

Nevertheless, as “Big Top” did not suffer severe chilling injury, biochemical and molecular parameters associated with these disorders as well as the rootstocks effects should be confirmed with peach cultivars more susceptible to chilling injury.

## 3. Materials and Methods

### 3.1. Plant Material

“Big Top” nectarine cultivar was budded in 2007 on seven *Prunus* rootstocks with different genetic background: three peach–almond hybrids (“Adarcias”, “GF 677” and “PADAC 9902-01”), one *P. persica* × *P. davidiana* hybrid (“Cadaman”), one hexaploid *P. insititia* plum (“Adesoto 101”), one plum x peach–almond hybrid (“PADAC 99-05”) and one plum–almond hybrid (“ReplantPAC”). Trees were established in an experimental orchard trial at EEAD-CSIC (41°43′42.7″ N, 0°48′44.1″ W, Zaragoza, Spain) during the winter of 2008-09 in a randomized complete block design with five replicates per tree. Trees were trained to a low-density open-vase system (5 m × 4 m). Standard pest, weed control and fertilization practices were implemented. Trees were drip-irrigated with a maximum dose of 250 m^3^ ha^−1^ week^−1^ during June and July, when water demands were highest. Crop load was adjusted per rootstock to avoid yield bias. Thus, hand-thinning was carried out at 40 days after full bloom (DAFB) to maintain a minimum spacing between fruits (approximately 20 cm). At harvest, all fruits per tree were weighted to determine total yield (kg/tree). The influence of these rootstocks on the agronomic characteristics of “Big Top” (tree vigor, yield, productivity, fruit quality and leaf mineral nutrition) was previously reported [11,13].

### 3.2. Fruit Sampling

For two consecutive years (2020 and 2021), forty representative fruits, with no sign of damage, were collected at harvest maturity (≈40 Newtons) per each tree replicate. Fruits were disinfected by immersion in aqueous solution of 5% sodium hypochlorite. Twenty fruits were analyzed at harvest and the remaining twenty fruits were stored at 5° during 28 days plus 2 days at room temperature (28 + 2 d). At harvest and after 28 + 2 d, composite samples for each biological replicate were taken from peeled fruits and from both halves. For further analysis, samples were frozen in liquid nitrogen, lyophilized and milled to powder in a M301 mill (Retsch, Dusseldorf, Germany, GmbH).

### 3.3. Chilling Injury Symptoms

After 28 days in the cold chamber, fruits were taken out and left at room temperature (25 °C) for 48 h (28 + 2 d). Fruits were cut into halves and chilling injury symptoms were evaluated according to browning (1 to 6), bleeding (1 to 6) and mealiness (1 to 3) following the scores stablished in the work by Lurie and Crisosto [3]. The higher the values, the higher the damage. Leatheriness was evaluated as the percentage of fruits presenting this symptom from the total (part per unit).

### 3.4. Fruit Quality Parameters

Basic fruit quality traits were determined as described by Font i Forcada et al. [13] including flesh firmness (FF), soluble solids (SSC) and titratable acidity (TA). Flesh firmness was measured using a penetrometer (FT-32), SSC was measured with a digital refractometer (Atago PR-101, Tokyo, Japan) and TA was determined in pulp using an automatic titration system with NaOH 0.1 M and pH end-point of 8.1.

### 3.5. Sugars and Organic Acid Profile

For sugars and organic acid extraction, 200 mg of lyophilized samples was mixed with 1.8 mL of methanol 80% and incubated during 1 h at 4 °C. Samples were centrifuged for 30 min at 4 °C and 12,000 rpm. Two hundred µL of methanol extracts were vacuum-concentrated (SPD111V SpeedVac, Thermo Fisher Scientific, Waltham, MA, USA, EEUU), resuspended with 800 µL of milli-Q water and filtrated to remove large particles. For the analysis, high-performance liquid chromatography (HPLC) was used. Sugars and organic acid profiles were analyzed using a column Rezex™ ROA Organic Acid H+ (8%) (300 mm × 7.8 mm, Phenomenex). Sugars were determined with a refractive index detector (Waters 2410, Waters Corporation, Milford, CT, USA) at 35 °C and acids with a photodiode array detector (Waters 2489, Milford, CT, USA) at 210 nm.  Mobile phase was a sulfuric acid solution (0.005 N), filtered and degassed, with a flow rate of 0.5 mL/min and at room temperature. Individual sugars and organic acids in sample extracts were identified and quantified by PC Millennium 3.2 software (Waters). Sugars and organic acid concentrations were expressed as g per kg of dry weight (DW).

### 3.6. Antioxidant Compounds

For antioxidant compounds (TPC, TFC and RAC) extraction, 200 mg of lyophilized samples was mixed with 1.8 mL of methanol 80% and incubated during 1 h at 4 °C. Vitamin C was extracted by mixing the lyophilized samples with HPO_3_ 5% (*v*/*v*) at 4 °C. Samples were centrifuged for 30 min at 4 °C and 12,000 rpm. Antioxidant compounds were analyzed using a 96-well microplate spectrophotometer photodiode array detector Asys UVM 340 microplate reader (Biochrom, Cambridge, UK) and the software DigiRead 1.8. Standard calibration curves were prepared daily on each microplate using eight different concentrations of gallic acid, catechin, trolox and ascorbic acid, for TPC, TFC, RAC and vitamin C, respectively. **Total phenolics content (TPC)** was determined as described in Singleton and Rossi [33] with modifications. Fifty μL of diluted extract (1:10) was mixed with 100 μL of Folin–Ciocalteau reagent 0.2 N. After 3 min of reaction at room temperature, 50 μL of Na_2_CO_3_ 12% (*v*/*v*) was added. Samples were kept 1 h in the dark at room temperature and absorbance was measured at 765 nm. Results are expressed in mg of gallic acid equivalents (GAEs) per g of DW. **Total flavonoids content (TFC)** was determined using a colorimetric assay according to Zhishen et al.’s [34] method. Forty μL of diluted methanolic extracts (1:1) was mixed with 50 μL of NaNO_2_ 5% (*w*/*v*). After 5 min at 30 °C and shaking (Thermo-Shaker PST-60HL, Biosan), 50 μL of AlCl_3_ 10% (*w*/*v*) was added. Then, after 5 min of incubation, 50 μL of NaOH 1 M was added. Absorbance was measured at 510 nm and the results are expressed in mg of cathequin equivalents (CEs) per g of DW. **Relative antioxidant capacity (RAC)** was determined according to Brand-Williams et al.’s [35] adapted method. Briefly, 20 μL of diluted methanolic extracts (1:10) was mixed with 200 μL of 2,2-diphenyl-1- picrylhydrazyl (DPPH) 80 mg/L and incubated 1 h in the dark at room temperature. Absorbance was measured at 515 nm and the results are expressed as μg trolox equivalents (TEs) per gram of DW. **Vitamin C** (**ascorbic acid, AsA) content** was estimated with Okamura’s method [36]. Forty μL of diluted metaphosphoric extracts (1:1) was mixed in the plate with 50 μL of H_3_PO_4_ 42.5% (*v*/*v*), 50 μL of 4% (*v*/*v*) bipyridyl in methanol: water (70%) and 50 μL of FeCl_3_ 1.2% (*v*/*v*). After 60 min of incubation in the Thermo-Shaker (37 °C, 500 rpm), absorbance was measured at 525 nm. Vitamin C was expressed in mg of ascorbic acid per g of DW. **Anthocyanin** content was determined according to a pH-differential method [37]. Fifty μL of methanolic extracts was mixed in the plate with 200 μL of potassium chloride buffer 0.025 M pH 1 and with sodium acetate buffer 0.4 M pH 4.5. Absorbance was read after 15 min at room temperature at 510 nm and 700 nm. The results are expressed as µg of Cyanidin-3-Glucoside Equivalents (C3GE) per g of DW using a molar extinction coefficient of ε = 26,900 (L·cm^−1^·mol^−1^).

### 3.7. PAL, PPO and POD Enzymatic Activities

Two hundred mg of lyophilized samples was mixed with 1.8 mL extraction buffer [PVP 1% (*w*/*v*), EDTA 0.5 M and Triton X-100 0.5%, pH 6.8] according to the work by Galeazzi et al. [38]. Samples were centrifuged at 4 °C for 30 min at 12,000 rpm. Protein content, POD and PPO activities were analyzed using a 96-well microplate spectrophotometer photodiode array detector Asys UVM 340 microplate reader (Biochrom, Cambridge) and PAL activity using a UV-2450 spectrophotometer (TCC-240A, Shimadzu, Kyoto, Japan). For **protein content** determination, the standard calibration curve was prepared on each microplate using eight different concentrations of Bovine Serum Albumin (BSA). Protein extracts were diluted 1:20 and 20 μL of diluted extract were added to each cell with 200 μL of Bradford reagent (1:4). Samples were incubated for 5 min at room temperature. The results were measured at 595 nm and expressed as mg BSA per g DW. **Phenylalanine ammonia lyase (PAL) activity** was determined according to the work by Tovar et al. [39]. Twenty μL of enzymatic extracts was mixed with 1 mL of 60 mM L-phenylalanine in 100 mM Tris-HCl pH 8. After incubation at 40 °C for 5 min, the reaction was stopped on ice. Cinnamic acid concentration was estimated by absorbance at 290 nm (ε = 17,400 L/cm·mmol). **Polyphenol oxidase (PPO) activity** was assayed according to the work by Galeazzi et al. [38]. Ten μL of enzymatic extract was mixed with 200 μL of 4-methylcathecol 10 mM in sodium acetate buffer 100 mM pH 5.5 and incubated 5 min at 35 °C. The PPO activity was estimated by the increase in absorbance at 420 nm. **Peroxidase (POD) activity** was determined according to the work by Dann and Deverall [40]. Ten μL of enzymatic extracts was mixed with 200 μL of substrate solution (guaiacol 20 mM with H_2_O_2_ 0.02%) in sodium acetate buffer 100 mM pH 5.5 and incubated 5 min at 30 °C. The POD activity was estimated by the increase in absorbance at 470 nm. PAL, PPO and POD enzymatic activities were expressed in units of enzymatic activity (U) per gram of protein. One unit was defined as an absorbance increase of 0.1 per minute for the current assay.

### 3.8. Gene Expression Analysis

Total RNA was isolated from lyophilized samples as previously reported [5]. The concentration of RNA was determined by an UV spectrophotometer (NanoDrop ND-2000, Thermo Fisher Scientific, Wilmington, DE, USA). The quality of RNA was confirmed by a 1% agarose gel stained with SYBR Safe (S33102, Thermo Fisher Scientific) and using Gel DOC 2000 (Biorad, Hercules, CA, USA) gel imager. DNA was digested by DNAsa I RNAsa-free (EN0521, Thermo Scientific) and reverse-transcribed using a First Strand cDNA synthesis kit (K1612, Thermo Scientific).

For real-time PCR (RT-PCR), five candidate reference genes were tested according to previous studies: *Translation Initiation Factor 1A* (*eIF1A*), the best reference gene according to Kou et al. [41]; and *Actin* (*ACT11*), *Translation Elongation Factor* (*TEF2*), *RNA polymerase II* (*RP II*) and *EST* (Gene Bank accession No. DY652828) according to Dos Santos Pereira et al. [42]. GeNorm (V. 3.5) tool was used to select the most stable candidate gene. NormFinder algorithm was used to identify the gene expression stabilities (M) of the five candidate reference genes. The gene with lower M value was considered as the most stable with a cut-off value of 0.15, as proposed by GeNorm program.

Amplifications were performed on Real Time PCR System (QuantStudio 3, Real-Time PCR System, Thermo Scientific) using specific primers for *PAL1*, *PPO4*, *POD2*, *CAT1*, *PME1*, *PG2*, *EXP3*, *CHI2* and *LDOX* genes (Table 3). Reactions included SYBR^®^ Green PCR Master Mix (A25777, Applied Biosystems). The PCR program used was as follows: 50 °C for 2 min and 95 °C for 10 min; 40 cycles at 95 °C for 15 s and 60 °C for 1 min; 95 °C for 15 s, 60 °C for 1 min and 95 °C for 1s. Efficiencies (E) and quantification cycles (Cq) values were determined using the LingRegPCR software 2012.3.0.0. and relative expression ratios (R) of target genes were calculated as described by Pfaffl [43].

### 3.9. Statistical Analysis

Means from each scion–rootstock combination were statistically analyzed by IBM SPSS Statistics 28.0.1.1 (Unites States) software. Shapiro–Wilk test was conducted to assess normality. Homoscedasticity was tested using Levene’s test. Factorial ANOVA analysis was conducted to determine the differences between treatments, rootstocks and years (2020 and 2021). The Duncan’s multiple range test (*p* ≤ 0.05) was performed for separation of means when F test was significant. Correlations between parameters were determined using the Pearson correlation coefficient at *p* ≤ 0.05. Principal component analysis (PCA) was performed to group the parameters of the study and identify the most interesting genotypes for our objectives.

## 4. Conclusions

These results improve the knowledge of nectarine performance budded on different rootstocks. A significant influence of the evaluated *Prunus* rootstocks was found concerning CI symptoms, basic fruit quality traits, sugars and organic acid profiles, antioxidants, enzymatic activities and relative expression of putative candidate genes. These findings demonstrate the importance of the rootstock on the harvest and post-harvest fruit quality. However, it is not possible to know whether the observed differences between harvest and 28 + 2 d are only attributable to the chilling treatment or to the subsequent shelf life.

Phenols, anthocyanins, enzymatic activities of PAL, POD and PPO as well as the *PAL1*, *PPO4*, *PG2* and *LDOX* genes’ expression were significantly correlated with chilling injury symptoms. Thus, these parameters could be important to consider in the prevention of chilling disorders. Moreover, some of these correlations were found to be significant regarding the harvest fruit values for these parameters, demonstrating the possibility to control CI alterations earlier, before shipping, to a certain extent.

Rootstocks with a plum genetic background induced, in general, higher vitamin C and antioxidant concentrations, especially “Adesoto 101” which could also increase the sweetness perception of the fruits. Consequently, these plum-based rootstocks could increase the ROS scavenging and health-promoting potential of the “Big Top” nectarine. However, higher antioxidant levels were also correlated with higher browning and bleeding symptoms.

Lower CI symptoms were found in rootstocks inducing lower antioxidant concentrations, such as “PADAC 9902-01”, “PADAC 99-05” or “ReplantPAC”. Therefore, the choice of these rootstocks will be more convenient for longer post-harvest periods and peach cultivars exhibiting the tendency to suffer higher CI. In contrast, for low-antioxidant cultivars such as “Big Top” where CI is not excessively severe, plum-based rootstocks such as “Adesoto 101” could be really interesting to obtain higher fruit quality.

Further work should be conducted to confirm rootstock influence on CI, with peach and nectarine cultivars being more susceptible to chilling injury.

## Figures and Tables

**Table 1:** Statistical significance of rootstock, treatment, year and their interaction on Big Top for quality parameters.

Trait	Variance Analysis							Variability (%)						
	T	R	Y	T*R	T*Y	Y*R	T*R*Y	T	R	Y	T*R	T*Y	Y*R	T*R*Y
Firmness (N)	***	ns	***	**	***	ns	ns	43.2	2.4	9.6	13.6	7.9	2.1	3.8
TA (mg MA/g DW)	***	ns	*	ns	ns	ns	*	45.7	12.5	4.5	7.3	3.3	5.7	13.3
SSC (mg SS/g DW)	*	ns	*	ns	ns	ns	ns	11.3	7.0	13.6	13.8	4.7	12.2	29.0
Sucrose (mg/g DW)	ns	ns	ns	ns	ns	ns	ns	2.8	5.9	3.8	30.4	2.5	33.8	22.4
Glucose (mg/g DW)	***	ns	***	ns	ns	ns	ns	53.8	11.4	13.1	13.2	0.5	5.6	3.2
Fructose (mg/g DW)	ns	*	***	ns	ns	***	ns	0.3	16.8	33.2	12.4	1.8	21.3	1.9
Sorbitol (mg/g DW)	***	ns	ns	ns	ns	ns	ns	47.1	1.1	3.6	13.8	0.6	14.0	10.6
Raffinose (mg/g DW)	***	**	***	**	ns	***	***	28.0	12.8	18.1	12.0	0.2	14.2	6.3
Myo-Inositol (mg/g DW)	ns	ns	***	ns	ns	ns	***	0.1	18.2	17.0	8.3	1.4	16.2	33.9
Galacturonic acid (mg/g DW)	**	*	***	ns	ns	ns	ns	17.8	25.5	12.9	14.6	0.0	12.1	10.0
Malic acid (mg/g DW)	***	*	ns	**	*	ns	ns	46.3	21.1	0.0	22.9	8.1	5.9	2.8
Quinic acid (mg/g DW)	**	***	***	*	**	ns	ns	6.8	32.0	13.7	22.9	2.3	6.0	8.8
Succinic + Shikimic acids (mg/g DW)	ns	***	ns	ns	ns	ns	ns	0.4	53.6	1.6	10.2	0.2	18.3	11.7
Citric acid (mg/g DW)	***	*	***	ns	*	ns	*	16.6	27.6	17.3	7.1	4.4	4.5	14.9
RAC (mg TE/g DW)	ns	***	***	ns	ns	*	ns	0.5	58.2	25.7	3.5	0.1	9.6	4.3
TPC (mg GAE/g DW)	ns	***	***	ns	ns	ns	ns	1.0	70.8	13.2	1.7	0.1	7.5	6.8
TFC (mg CAT/g DW)	ns	***	***	ns	ns	**	ns	0.8	40.7	38.0	1.3	0.1	11.4	4.3
AC (µg C3GE/g DW)	*	***	ns	ns	ns	ns	ns	8.1	56.7	1.2	9.5	2.3	11.5	6.8
AsA (mg AsA/g DW)	***	***	**	*	ns	ns	ns	56.9	17.9	4.8	8.8	0.4	3.6	3.9
Protein (mg BSA/g DW)	ns	***	***	ns	ns	ns	ns	0.8	25.1	33.1	4.5	0.0	11.9	3.5
PAL (U/g prot)	ns	**	***	ns	ns	ns	ns	3.5	31.4	25.6	4.9	0.0	18.3	4.9
POD (U/g prot)	ns	ns	***	ns	*	ns	*	2.7	9.1	55.9	13.5	0.1	4.1	12.8
PPO (U/g prot)	ns	*	***	ns	ns	ns	ns	0.5	30.4	38.1	3.5	0.9	9.0	9.5

Data were evaluated by three-way variance analysis (ANOVA) on raw data from 2020 and 2021 seasons at harvest and after 28 days plus 2 days of shelf life. Significance: * indicates *p* ≤ 0.05, ** *p* ≤ 0.01, *** *p* ≤ 0.001 and ns *p* > 0.05. N, Newtons. T, Treatment (harvest and 28 days at 5 °C + 2 days of shelf life). R, Rootstock. Y, Year. MA, Malic acid. DW, Dry Weight. TA, Titratable Acidity. SSC, Soluble Solids Content. DW, Dry Weight. RAC, Relative Antioxidant Capacity. TE, Trolox Equivalents. TPC, Total Phenolic Content. GAE, Gallic Acid Equivalent. TFC, Total Flavonoids Content. CAT, Catechin. AC, Anthocyanins content. C3GE, Cyanidin-3-Glucoside Equivalents. AsA, Ascorbic Acid. BSA, Bovine Serum Albumin. PAL, Phenylalanine ammonia lyase. U, Units. Prot, Protein. POD, Peroxidase. PPO, Polyphenol oxidase.

**Table 2:** Statistical significance of rootstock, treatment, year and their interaction on “Big Top” for gene-relative expressions.

Gene	Variance Analysis							Variability (%)						
	T	R	Y	T*R	T*Y	Y*R	T*R*Y	T	R	Y	T*R	T*Y	Y*R	T*R*Y
*Phenylalanine Ammonia Lyase 1 (PAL1)*	ns	*	ns	ns	ns	ns	ns	5.2	16.9	9.8	7.7	1.1	25.8	12.8
*Polyphenol Oxidase 4 (PPO4)*	*	ns	ns	ns	ns	**	ns	0.6	35.3	4.1	1.3	0.5	34.4	1.4
*Peroxidase 2 (POD2)*	ns	**	ns	ns	ns	ns	ns	1.1	18.3	6.8	17.3	10.7	25.0	11.8
*Catalase 1 (CAT1)*	*	***	***	ns	ns	***	ns	2.6	45.9	17.1	7.5	1.1	20.1	7.5
*Pectin Methylesterase 1 (PME1)*	ns	**	ns	ns	ns	ns	ns	2.1	39.9	2.1	13.7	1.0	18.6	4.5
*Polygalacturonase 2 (PG2)*	***	**	**	ns	***	ns	ns	69.8	6.6	3.5	2.4	5.4	4.3	2.7
*Expansin 3 (EXP3)*	*	ns	***	*	ns	*	*	0.2	12.9	16.5	22.4	2.9	19.4	21.4
*Chalcone Synthase 2 (CHI2)*	***	ns	***	ns	ns	ns	ns	25.3	3.4	23.5	3.3	3.9	15.4	5.1
*Leucoanthocyanidin Dioxygenase (LDOX)*	ns	ns	ns	ns	ns	ns	ns	3.1	19.2	4.2	10.6	1.2	28.2	15.3

Data were evaluated by three-way variance analysis (ANOVA) on raw data from 2020 and 2021 seasons at harvest and after 28 days plus 2 days of shelf life. Significance: * indicates *p* ≤ 0.05, ** *p* ≤ 0.01, *** *p* ≤ 0.001 and ns *p* > 0.05. T, Treatment (harvest and 28 days at 5 °C + 2 days of shelf life). R, Rootstock. Y, Year.

**Table 3:** Primer sequences used for qPCR analysis.

GDR or NCBI Accession No	Gene Name	Primer Sequence 5′-3′		Source
JC687766	*Phenylalanine Ammonia Lyase 1*	F R	CAGAGCAGCACAACCAAGACG CTCCAAATGCCTCAAATCAATG	[44]
XM_020561913	*Polyphenol Oxidase 4*	F R	CAAATGCGGCAGAGACCTCAAA CTTCCTTCTCCTTCTGGCTCCT	[32]
DW352089	*Peroxidase 2*	F R	CGGTTTGGTGTACTTTGCGATCG TCATTTATTCATACAGAGCTGGC	[45]
AJ496418	*Catalase 1*	F R	GGATGCCCTATCAGACCCAC TAATCCCAAATGACAATCCG	[46]
ppa003852m	*Pectin Methylesterase 1*	F R	CAATCATCTATGTCAAGGAAGG CCAGCCATCAACTACACTT	[8]
ppa006857m	*Polygalacturonase 2*	F R	ACAATCCTCAACTCCAAGA AACGCCTTCTATCCACAA	[8]
ppa010180m	*Expansin 3*	F R	GGGTTGGTGTGATTTTGTGAG AGTATTTATAGGGTGCGGGCTAC	[9]
AB094986	*Chalcone Synthase 2*	F R	CCCATCATCCGCTTCAT CCCAGGTTCCCATCTTGT	[47]
ppa007738m	*Leucoanthocyanidin Dioxygenase*	F R	TACCCTGAGGACAAGCGTGAC ATCCCAACCCAAGTGACAGC	[44]

## Data Availability

Data are contained within the article and supplementary materials.

## Supplementary Materials

The following supporting information can be downloaded at: https://www.mdpi.com/article/10.3390/plants13050677/s1, Table S1: Basic fruit quality means and significance for the treatment and rootstock main factors; Table S2. Individual sugar means and significance for the treatment and rootstock main factors; Table S3. Organic acid means and significance for the treatment and rootstock main factors; Table S4. Antioxidant means and significance for the treatment and rootstock main factors; Table S5. Enzymatic activity means and significance for the treatment and rootstock main factors.
